# Molecular Phylogeny and Taxonomy of the Butterfly Subtribe Scolitantidina with Special Focus on the Genera *Pseudophilotes*, *Glaucopsyche* and *Iolana* (Lepidoptera, Lycaenidae)

**DOI:** 10.3390/insects13121110

**Published:** 2022-11-30

**Authors:** Vladimir A. Lukhtanov, Anastasia V. Gagarina

**Affiliations:** Department of Karyosystematics, Zoological Institute, Russian Academy of Sciences, Universitetskaya Nab. 1, 199034 Saint-Petersburg, Russia

**Keywords:** Lepidoptera, Lycaenidae, Polyommatini, host plant, phylogeny, DNA barcoding

## Abstract

**Simple Summary:**

The Palearctic butterfly genera *Pseudophilotes*, *Glaucopsyche* and *Iolana* have attracted the attention of many entomologists because their species are used as model objects for studying ecology and evolution. The genera have previously been the subjects of several taxonomic studies based on the analysis of their morphological and molecular characteristics, but none of these studies are based on complete species sampling. In our work, we used a set of mitochondrial and nuclear genes to reveal the phylogeny of these genera as well as the phylogeny of the subtribe Scolitantidina, to which these genera belong. In the genus *Pseudophilotes*, we identified 10 species including among them, the enigmatic Central Asian taxon *P. panope*, which has often been assigned to other genera. We clarified the taxonomic structure of the genus *Glaucopsyche*, which was found to consist of four subgenera. We confirm that the genus *Iolana* includes nine species distributed across the southwestern part of the Palearctic. The results obtained here will be important for the conservation of the Scolitantidina species, some of which are local and protected by national and international laws.

**Abstract:**

The Palearctic blue butterfly genus *Pseudophilotes* Beuret, 1958 is not homogenous regarding the morphology of its genital structures. For this reason, some of its species have been considered to be representatives of other genera of the subtribe Scolitantidina (subfamily Polyommatinae). Here, we address these taxonomic problems by analyzing the phylogenetic relationships between the genera, subgenera, and species of this subtribe inferred via the analysis of five nuclear and two mitochondrial DNA sequences. We demonstrate that the enigmatic Asian species *P. panope* (Eversmann, 1851) belongs to the genus *Pseudophilotes* but not to *Praephilotes* Forster, 1938 or *Palaeophilotes* Forster, 1938 and does not represent the independent genus *Inderskia* Korshunov, 2000, as hypothesized previously. We synonymize *P. svetlana* Yakovlev, 2003 (**syn. nov**.) and *P. marina* Zhdanko, 2004 (**syn. nov**.) with *P. panope*. We demonstrate a deep genetic divergence between lineages that were previously considered as subspecies of the single species *Iolana iolas* (Ochsenheimer, 1816). As a result, we confirm the multispecies concept of the genus *Iolana* Bethune-Baker, 1914. We show that the Holarctic genus *Glaucopsyche* can be divided into four subgenera: *Glaucopsyche* Scudder, 1872 (=*Shijimiaeoides* Beuret, 1958), *Apelles* Hemming, 1931, *Bajluana* Korshunov and Ivonin, 1990, and *Phaedrotes* Scudder, 1876.

## 1. Introduction

The subtribe Scolitantidina Tutt, 1907 belongs to the tribe Polyommatini (subfamily Polyommatinae) and includes about 17–22 genera and about 85–100 described species distributed throughout the Holarctic and Oriental regions [1]. Eliot [2] recognized this group as a cluster of morphologically similar genera and called it “the *Glaucopsyche* section” (after the name of one of the genera in this group). Mattoni [3] treated it as the tribe Scolitantidini. Hesselbarth et al. [4] divided this group into the subtribes Scolitantidina and Glaucopsychina Hemming, 1931 within the tribe Polyommatini. Subsequent studies confirmed the monophyly of this group, but the division into the subtribes Scolitantidina and Glaucopsychina was not supported [5,6]. Over the past 50 years, this subtribe has been the subject of a series of taxonomic and phylogenetic studies based on the use of morphological and molecular markers [3,5,6,7,8,9]. Despite this, the phylogenetic position, taxonomic status (genus-subgenus-synonymy) and species diversity of some genera within the subtribe Scolitantidina have remained unclear. In particular, this applies to the genera *Pseudophilotes*, *Glaucopshyche* and *Iolana*. 

The genus *Pseudophilotes* was found not to be homogenous regarding the morphology of its genital structures and larval food plants [10,11,12,13,14,15]. For this reason, some of its species have been considered to be representatives of other genera, including *Rubrapterus* [10,11], *Inderskia* [12], *Praephilotes* [13], and *Palaeophilotes* [14,15]. The genus Pseudophilotes includes somewhere between eight and twelve species that are distributed across the temperate zone of Eurasia from the Atlantic coast in the west to East Siberia (Yakutia) in the east, and are also found locally in North Africa and the Levant [16]. This genus has attracted the attention of numerous researchers, as some of its species have been used as models in ecological [17,18,19,20] and evolutionary studies [16,21], particularly in studies of insect–plant coevolution [22,23]. Nearly all species of the genus are considered endangered or threatened and are protected by international and/or national laws [24,25,26,27,28,29,30,31,32,33]. The genus *Pseudophilotes* has been the subject of several taxonomical studies based on analyses of its morphology [4,7,10,12,14,15,34,35,36] and molecular markers [6,9,16,20,21,37]. The morphological analyses revealed an unusually high level of male genitalia variations on both intra- and inter-specific levels, resulting in descriptions of several new taxa [10,12,14,34,35,36]. Available multilocus molecular studies have focused on particular species and species groups within the genus *Pseudophilotes* [16,20,21], but none of them are based on complete species sampling. In particular, no information on molecular markers is available for the Asian species *P. panope*, *P. svetlana*, and *P. marina*. The genus *Glaucopshyche* was revealed to be a paraphyletic entity in a phylogenomic study by Ugelvig et al. [6] and a monophyletic group in a whole-genome study by Zhang et al. [9]. However, both studies [6,9] were based on an incomplete sampling of nominal genera and did not include the taxon *Bajluana* Korshunov and Ivonin, 1990, which is based on the little-known and morphologically distinct species, *Glaucopsyche argali*.

The genus *Iolana* is distributed throughout countries surrounding the Mediterranean Sea, in the Levant, Iran, Central Asia, northern Pakistan and northern India. It is represented by a number of allopatric, clearly closely related, but morphologically well-differentiated taxa. These taxa are considered as (i) subspecies of the same species, (ii) representatives of the two species *I. iolas* and *I. gigantea*, or (iii) seven to nine independent species [4,13]. These three taxonomic hypotheses have never been tested using molecular markers. 

Here, we address these taxonomic problems by analyzing the phylogenetic relationships between the species of the subtribe Scolitantidina inferred via an analysis of the nuclear genes *ribosomal subunit 28S* (*28S*), *histone 3* (*H3*), *elongation factor 1α* (*EF1α*) and *wingless* (*wg*), the non-coding nuclear *internal transcribed spacer 2* (*ITS2*), and two mitochondrial genes, *cytochrome oxidase I* and *II* (*COI* and *COII*).

## 2. Materials and Methods

### 2.1. Taxon Sampling

According to Eliot [2], Mattoni [3], Ugelvig et al. [6], and Korshunov and Ivonin [36], the following genera should be included in the subtribe Scolitantidina (=*Glaucopsyche* section sensu Eliot, 1973): 

*Apelles* Hemming, 1931 (Type-species [TS]: *Polyommatus melanops* Boisduval, [1828]);

*Bajluana* Korshunov & Ivonin, 1990 (TS: *Lycaena argali* Elwes, 1899);

*Caerulea* Forster, 1938 (TS: *Lycaena coeligena coelestis* Alpheraky, 1897);

*Euphilotes* Mattoni, [1978] (TS: *Lycaena enoptes* Boisduval, 1852);

*Glaucopsyche* Scudder, 1872 (TS: *Polyommatus lygdamus* Doubleday, 1841);

*Inderskia* Korshunov, 2000 (TS: *Lycaena panope* Eversmann, 1851);

*Iolana* Bethune-Baker, 1914 (TS: *Lycaena iolas* Ochsenheimer, 1816);

*Maculinea* van Ecke, 1915 (TS: *Papilio alcon* Denis & Schiffermüller, 1775);

*Micropsyche* Mattoni 1981 (TS: *Micropsyche ariana* Mattoni, 1981);

*Otnjukovia* Zhdanko [1997] (TS: *Turanana tatjana* Zhdanko, 1984);

*Palaeophilotes* Forster, 1938 (TS: *Lycaena triphysina* Staudinger, 1892);

*Phaedrotes* Scuder, 1876 (TS: *Lycaena catalina* Reakirt, 1866, currently a subspecies of *Lycaena piasus* Boisduval, 1852);

*Phengaris* Doherty, 1891 (TS: *Lycaena atroguttata* Oberthür, 1876);

*Philotes* Scudder, 1876 (TS: *Lycaena regia* Boisduval; 1869 currently subspecies of *Lycaena sonorensis* C. & R. Felder, [1865]);

*Philotiella* Mattoni, [1978] (TS: *Lycaena speciosa* H. Edwards, [1877]);

*Praephilotes* Forster, 1938 (TS: *Lycaena anthracias* Christoph, 1877);

*Pseudophilotes* Beuret, 1958 (TS: *Papilio baton* Bergsträsser, [1779]);

*Rubrapterus* Korshunov, 1987 (TS: *Lycaena bavius* Eversmann, 1832);

*Scolitantides* Hübner, 1819 (TS: *Papilio battus* Denis & Schiffermüller, 1775; currently a subspecies of *S. orion* Pallas 1771);

*Shijimiaeoides* Beuret, 1958 (TS: *Lycaena barine* Leech, 1893; currently subspecies of *S. divina*);

*Sinia* Forster, 1940 (TS: *Glaucopsyche* (*Sinia*) *leechi* Forster, 1940);

*Subsolanoides* Koiwaya, [1989] (TS: *Subsolanoides nagata* Koiwaya, 1981);

*Turanana* Bethune-Backer, 1916 (*TS: Lycaena cytis* Christoph, 1877).

**Figure 1:**
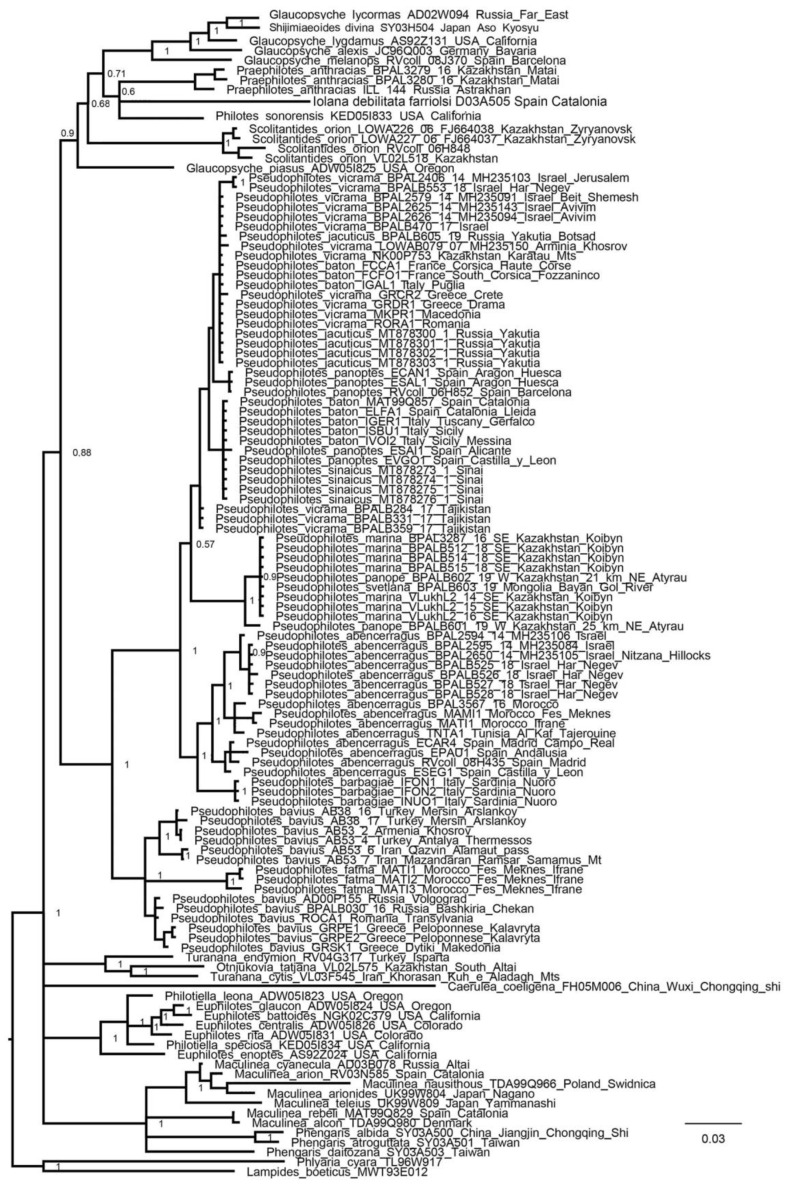
BI tree of the subtribe Scolitantidina based on mitochondrial genes (*COI +COII* dataset). Posterior probabilities are indicated at the nodes.

**Figure 2:**
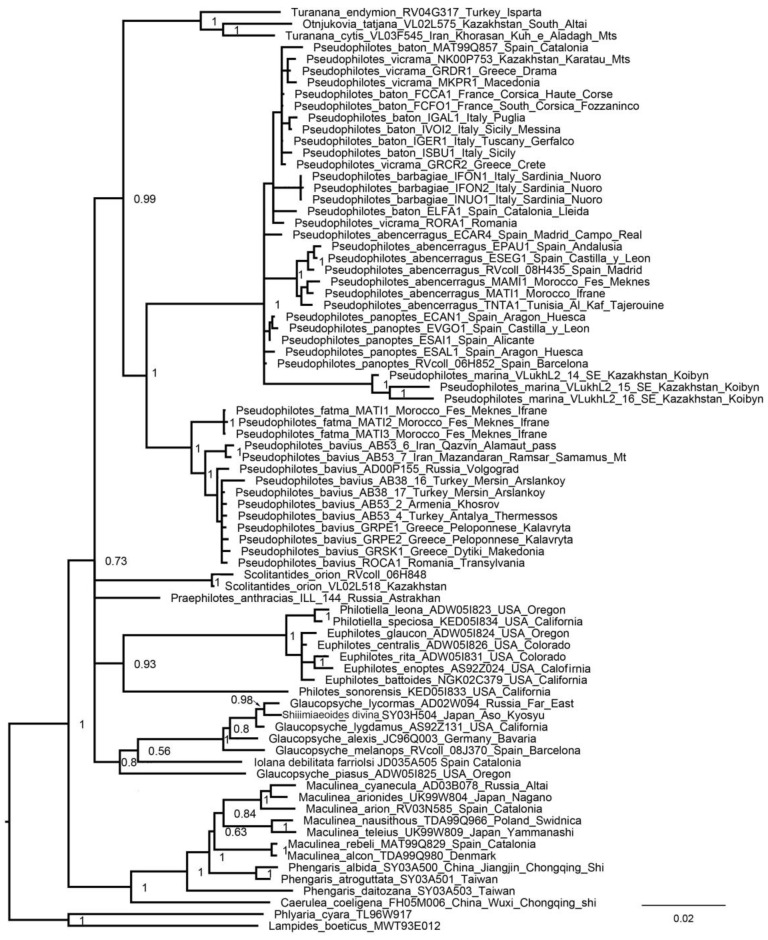
BI tree of the subtribe Scolitantidina based on nuclear sequences (*28S + H3 + EF1-a + wingless + ITS2* dataset). Posterior probabilities are indicated at the nodes.

**Figure 3:**
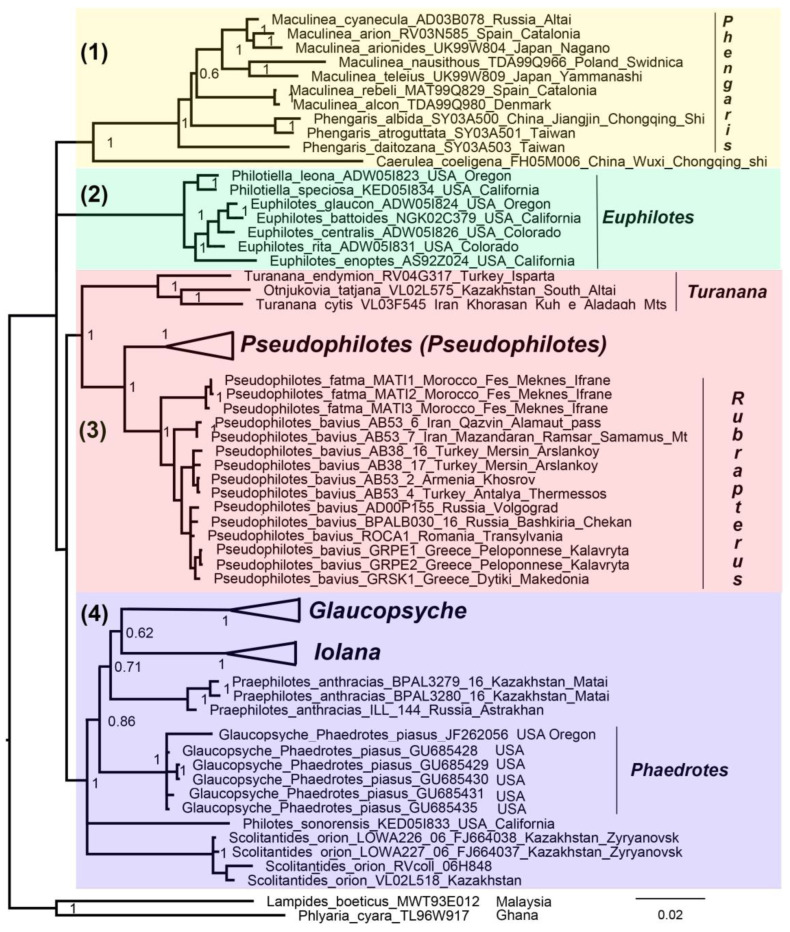
BI of the subtribe Scolitantidina based on concatenation of nuclear and mitochondrial genes (*28S* + *H3* + *EF1-a* + *wingless + ITS2 + COI + COII* dataset). Posterior probabilities are indicated at the nodes. (1), (2), (3), and (4) are the four supported main lineages within the subtribe Scolitantidina.

**Figure 4:**
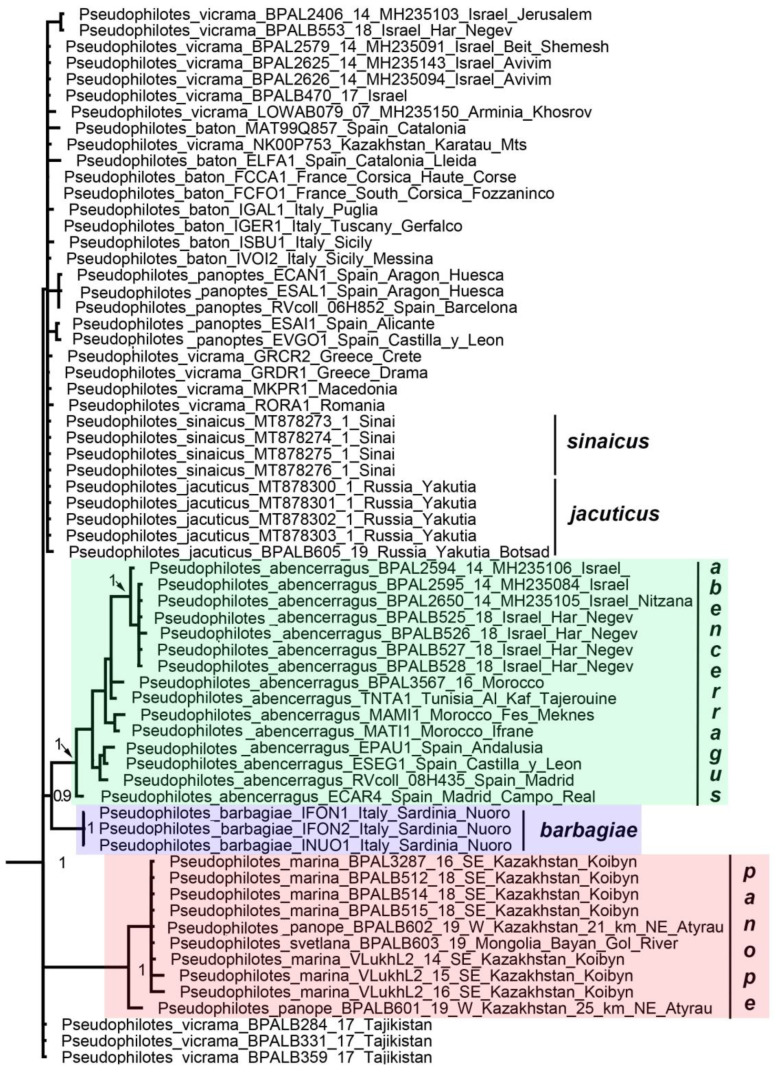
Fragment of the concatenated (*28S* + *H3* + *EF1-a* + *wingless + ITS2 + COI + COII*) BI tree. The subgenus *Pseudophilotes* (*Pseudophilotes*) (=*Inderskia*, **syn. nov**.) is shown. Posterior probabilities are indicated at the nodes.

**Figure 5:**
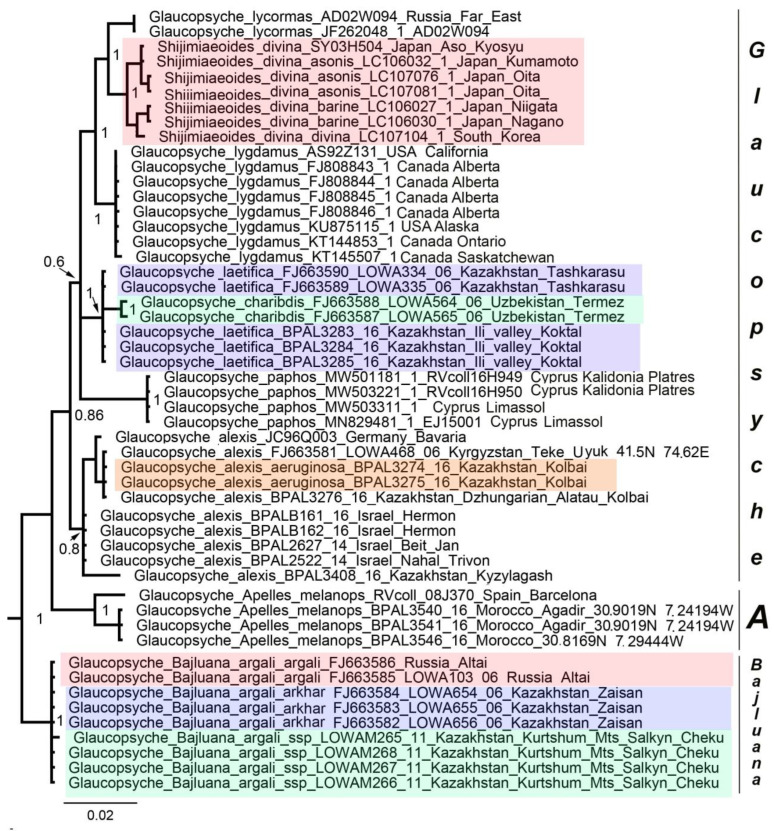
Fragment of the concatenated (*28S* + *H3* + *EF1-a* + *wingless + ITS2 + COI + COII*) BI tree. The genus *Glaucopsyche* is shown. A is the subgenus *Apelles*. Posterior probabilities are indicated at the nodes.

**Figure 6:**
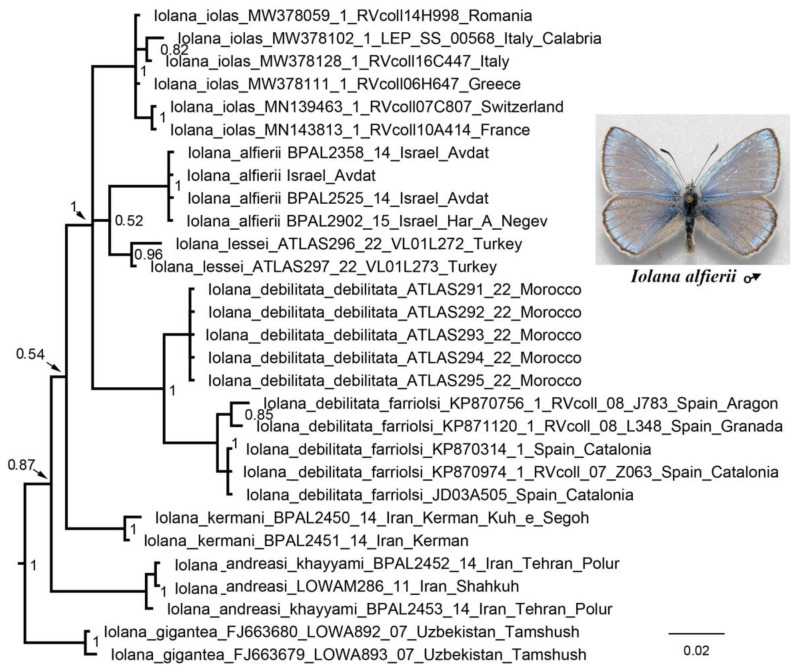
Fragment of the concatenated (*28S* + *H3* + *EF1-a* + *wingless + ITS2* + *COI + COII*) BI tree. The genus *Iolana* is shown. Posterior probabilities are indicated at the nodes.

For molecular analysis, we used representatives of all these nominal genera, except the very rare monotypic Central Asian genera *Palaeophilotes*, *Micropsyche*, *Sinia* and *Subsolanoides*. The species sampling included the type species for all studied nominal genera. For the genus *Pseudophilotes*, we analyzed representatives of all traditionally recognized species. The GenBank and/or BOLD accession numbers of the studied samples are presented in Table 1 and Figure 1, Figure 2, Figure 3, Figure 4, Figure 5 and Figure 6. These accession numbers are searchable via GenBank (https://www.ncbi.nlm.nih.gov/genbank/, accessed on 28 November 2022) and/or BOLD (https://boldsystems.org/index.php/Public_BINSearch?searchtype=records, accessed on 28 November 2022) sites that contain information about the sequences and vouchers.

### 2.2. DNA Studies

The nuclear DNA sequences *28S*, *ITS2*, *EF1-a* and *wg* were obtained from the department of Karyosystematics (Zoological Institute RAS, St. Petersburg) using the primers and protocols described in [16]. Standard *COI* barcodes (partial sequences of the *cytochrome c oxidase subunit I* gene) were obtained from the Canadian Centre for DNA Barcoding (CCDB, Biodiversity Institute of Ontario, University of Guelph) using their standard high-throughput protocol described by deWaard et al. [38]. The pictures, and collection data of these specimens have been deposited and can be freely downloaded from the BOLD Public Data Portal (http://www.boldsystems.org/index.php/databases, accessed on 28 November 2022). Information about the obtained sequences is presented in Table 1.

For the analyses we used our own sequences as well as published sequences (nuclear sequences *28S*, *H3*, *EF1-a*, *wingless*, and *ITS2* and mitochondrial genes *COI* and *COII*) extracted from GenBank [6,9,16,20,21,39,40,41,42,43] (Table 2). The GenBank/BOLD/museum accession numbers of the analyzed sequences are presented in Figure 1, Figure 2, Figure 3, Figure 4, Figure 5 and Figure 6. Two taxa (*Lampides boeticus* and *Phylaria cyara*) belonging to the *Lampides* and *Phylaria* sections sensu Eliot, 1973 were used to root the tree. The nuclear ribosomal *28S rRNA* gene fragment and the nuclear *ITS2* sequences were aligned with the software MAFFT v7.245, using the iterative refinement method G-INS-i [44] via the MAFFT online server (http://mafft.cbrc.jp/alignment/server/, accessed on 28 November 2022). As the *ITS2* region consists of highly variable sections, its alignment remained partly ambiguous. We therefore used the software Aliscore v.2.0 (The Leibniz Institute for the Analysis of Biodiversity, Bonn, Germany) [45] to identify the ambiguously aligned or randomly similar sections within the *ITS2* alignment as described previously [16]. Other sequences were aligned using BioEdit software [46] and were edited manually. Nucleotide substitution models for each dataset were estimated based on the Bayesian information criterion using jModeltest, version 2 [47]. The best fitting models were as follows: GTR + G + I for *28S*, GTR + G + I for *COI*, GTR + G for *H3*, K2 + G + I for *EF1a*, K2 + G for *wg*, GTR + G + I for *COII* and K2 + G for *ITS2*.

The Bayesian analyses (Bayes inference, BI) were performed for each individual data set (*28S*, *COI*, *H3*, *EF1-a*, *wg*, *COII*, and *ITS2*) using the program MrBayes 3.2 [48] and the best fitting models. Two runs of 10,000,000 generations with four chains (one cold and three heated) were performed. The consensus of the obtained trees was visualized using FigTree 1.3.1 (http://tree.bio.ed.ac.uk/software/figtree/, accessed on 28 November 2022). These analyses revealed no significant gene tree–species tree conflicts in the data. Then, the genes were concatenated. In doing so, we were based on the evidence that combining multiple mitochondrial DNA barcodes with multilocus nuclear data for representative major taxa can significantly improve the resolution of phylogenetic analysis [49]. The concatenated alignment is presented in the Supplementary Materials (Table S1). The BI analysis of the concatenation 28S + *COI* + H3 + *EF1-a* + *wg* + COII + ITS2 was performed using partition of the data by gene.

### 2.3. Genus and Subgenus Concepts

We have previously argued that a genus-rank taxon must meet four criteria: (1) monophyly, (2) morphological discreteness, (3) conformity to a certain evolutionary age interval, and (4) conformity to historical nomenclatural traditions (stability and preservation of traditionally recognized taxa) [50]. While the first, second, and fourth criteria seem to be almost universally accepted, the use of criterion three (correspondence of the genus to a certain evolutionary age) is less obvious, and the evolutionary ages of traditionally accepted genera in different groups of living organisms vary greatly. Therefore, in this paper, we used three parameters as a genus criterion. Two of them are obligatory: (1) the monophyly and (2) the morphological discreteness from other genera. One was optional (the group was traditionally considered as a genus). We interpreted the existence of reasonable doubts about the monophyly of a genus as being in favor of dividing the group into two or more undoubtedly monophyletic entities.

As a subgenus, we considered a lineage that was also monophyletic and morphologically discrete but for which there was no tradition to consider it as a genus. Usually such a lineage (subgenus) in combination with other lineages (subgenera) forms a traditionally accepted genus. An additional (though not obligatory) reason for giving a lineage the status of a subgenus was the presence of a previously described available name for it.

### 2.4. Methodology of Molecular Taxonomy: Genomics, Phylogenomics, DNA-barcoding, and Mixed (Phylogenomics + Barcoding) Approaches

We live in a time when works based on genome-wide data are beginning to appear in insect taxonomy [e.g., 8,9], but at the same time, articles based on multilocus data (phylogenomic approach) [e.g., 6,16,20] or on single mitochondrial gene *COI* (DNA-barcoding approach) [39,40] still dominate. It seems to us that in between these methodologies is the mixed approach proposed by Talavera et al. [49] who demonstrated that DNA barcodes combined with multilocus data of representative taxa could generate reliable, higher-level phylogenies. This approach is indispensable for poorly studied groups and allows us to combine the suitable length of concatenated sequences for representative (“skeleton”) taxa with the completeness of species sampling.

## 3. Results

### 3.1. Mitochondrial Tree

Phylogenetic trees based exclusively on mitochondrial genes performed poorly, resulting in numerous polytomies (Figure 1). However, there were nodes that had good support. Thus, within the genus *Glaucopsyche*, the clade *Glaucopsyche lycormas* + *Shijimiaeoides divina* was identified. The genus *Pseudophilotes* was monophyletic and divided into two subgenera *Pseudophilotes* sensu stricto and *Rubrapterus.* The *P. panope* complex of the genus *Pseudophilotes* (*P. panope + P. marina + P. svetlana*) was monophyletic and isolated from other species of the genus. *Pseudophilotes abencerragus* and *P. barbagiae* were sister species. 

### 3.2. Nuclear Tree

On the nuclear tree (Figure 2), the clades representing the genera *Turanana*, *Phengaris* + *Caerulea*, and *Glaucopsyche* (including *Shijimiaeoides divina*) received good support. The genus *Pseudophilotes* was found to be monophyletic and divided into two subgenera *Pseudophilotes* sensu stricto and *Rubrapterus.* The subgenus *Rubrapterus* was found to include two monophyletic species: *P*. (*R*.) *fatma* and *P*. (*R*.) *bavius*. The nuclear data supported the monophyly of the subgenus *Pseudophilotes*; however, within this subgenus the phylogeny was not resolved. Only three species of the subgenus *Pseudophilotes* were found to be supported monophyletic entities (*P. abencerragus*, *P. marina* and *P. barbagiae*).

### 3.3. Concatenated Tree 

No serious topology conflict was found between the mitochondrial and nuclear trees. Therefore, the mitochondrial and nuclear data were combined resulting in a mixed matrix [49], in which both the DNA barcodes of multiple species and specimens and the multilocus data of representative taxa were represented (Table S1). This led to a noticeable increase in the resolution of the resulting phylogram (Figure 3). The following genera and suprageneric groups were identified as monophyletic: *Phengaris* (including *Maculinea*), *Phengaris* + *Caerulea*, *Philotiella*, *Euphilotes*, *Philotiella* + *Euphilotes* (Figure 3) *Turanana* (including *Otnjukovia*), *Pseudophilotes* (Figure 4), *Turanana* + *Pseudophilotes* (Figure 3), *Glaucopsyche* (Figure 5), *Iolana* (Figure 6), *Praephilotes*, *Phaedrotes*, and *Scolitantides* (Figure 3). 

## 4. Discussion

Our analysis revealed four supported main lineages within the subtribe Scolitantidina: (1) *Phengaris* + *Caerulea*; (2) *Euphilotes* + *Philotiella*; (3) *Pseudophilotes* + *Turanana*, and (4) *Scolitantides* + *Philotes* + *Praephilotes* + *Iolana* + *Glaucopsyche*. This result is consistent with the previous molecular data [6] but does not support the division of the studied group into the subtribes Scolitantidina and Glaucopsychina [4]. Within lineage (1) we found a pattern that was previously [5,6,7,51] described: the nominal genus *Maculinea* was nested within the genus *Phengaris*. The genus *Phengaris* (including *Maculinea*) was a sister of *Caerulea*. Within lineage (2), the sublineages *Euphilotes* and *Philotiella* were found to be closely related and weakly differentiated taxa. *Euphilotes* and *Philotiella* were described by Mattoni as two distinct genera [3]. Zhang et al. [8] downgraded *Philotiella* to the rank of a subgenus of *Euphilotes* because their *COI* barcodes differed by only 3.3%. Our data also showed that *Philotiella* was better treated as a subgenus than a genus. 

Lineage (3) included two sister genera: *Turanana* and *Pseudophilotes* (6, 9, our data). Phylogenomic data for *Otnjukovia* [5,6] and genomic data for *Micropsyche* [9] demonstrated that these taxa were junior subjective synonyms of *Turanana*.

The genus *Pseudophilotes* is divided into two subgenera: *Pseudophilotes* sensu stricto and *Rubrapterus*. Within the subgenus *Pseudophilotes*, one of the most controversial points is the phylogenetic position of the species *P. barbagiae*, endemic to Sardinia. In the work of Todisco et al. [37] and Bartoňová et al. [21], it was shown that, according to mitochondrial data, this was a sister species of *P. abencerragus*, which is distributed across North Africa, the Iberian Peninsula, and the Levant. At the same time, according to the combined nuclear–mitochondrial data [16], *P. barbagiae* was found to be included in the same clade as the European species *P. panoptes* and *P. baton*. Our analysis, as well as the data of Wiemers et al. [52], tends to support the sister relationship between *P. barbagiae* and *P. abencerragus*. The position of *P. barbagiae* on the phylogenetic tree is essential for deciding whether the species originated from Africa or from Europe, but it should be recognized that this issue has not yet been resolved. In the situation of apparent mitonuclear discordance, genome-wide data may be needed to resolve this problem.

*Pseudophilotes panope,* described by E. Eversmann from NW Kazakhstan, is one of the rarest and most enigmatic species of the subtribe Scolitantidina. Researchers previously attributed it to the genera *Pseudophilotes*, *Praephilotes*, *Paleophilotes*, *Inderskia*, or considered it as a species whose genus was unknown [3,53]. The obtained nuclear and mitochondrial molecular data indicated the undoubted proximity of this taxon to species of the subgenus *Pseudophilotes* (*Pseudophilotes*), resulting in the synonymy: *Pseudophilotes* Beuret, 1958 (=*Inderskia* Korshunov, 2000, **syn. nov**.). *Pseudophilotes panope* has long been known in western Kazakhstan [15] and has only recently been found in eastern Kazakhstan (described as *Paleophilotes* [sic] *marina* Zhdanko, 2004) and Mongolia (described as *Pseudophilotes svetlana* Yakovlev, 2003). A detailed analysis of the external morphology, male genitalia and ecological preferences of populations belonging to *P. panope*, *P. marina* and *P. svetlana* was carried out by Morgun [15]. This author concluded that “all populations are the forms of one species with slightly different phenotypes, which may be due to adaptation (e.g., color, type of soil in inhabited biotopes, altitude above sea level)”. Tshikolovets et al. [53,54] downgraded *P. marina* and *P. svetlana* to subspecies of *P. panope*. Our study revealed identical DNA barcodes in the populations from west and east Kazakhstan and Mongolia. Based on this, we propose a synonymy: *P. panope* Eversmann, 1851 (=*svetlana* Yakovlev, 2003, **syn. nov.**; =*marina* Zhdanko, 2004, **syn. nov.**).

**Figure 7:**
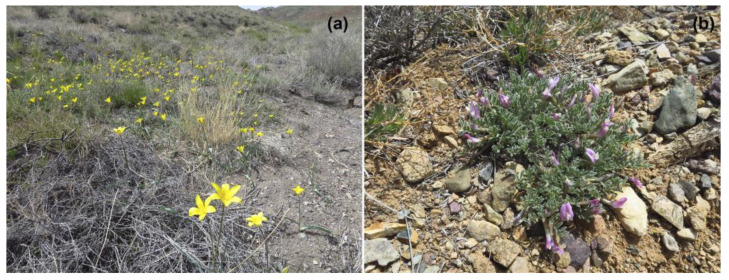
Habitat (**a**) and larval foodplant (*Astragalus lasiophillus*) (**b**) of *P. panope* in Dzhungarian Alatau, E. Kazakhstan. Photo: V.Lukhtanov.

An interesting feature of *P. panope* is its monophagy on *Astragalus lasiophillus* Ledebur (Fabaceae), whereas caterpillars of other species of the genus are predominantly associated with the plants of the family Lamiaceae [3,4,5,6,7,8,14,15,16,17,18,19,20,21,22]. Association with *Astragalus lasiophillus* has been also confirmed by us for the east Kazakhstan population of the species via observation of oviposition (Figure 7). A possible clue to this unusual feature is that another species of the genus, *P. abencerragus*, can also facultatively feed on plants of the family Fabaceae [22]. Feeding on legumes (Fabaceae) is probably an ancestral trait of Polyommatini butterflies [22]; this trait was either lost in the *Pseudophilotes* lineage but reappeared in *P. panope* as a reversion, or it was maintained in *P. panope* when the ancestor of the remaining *Pseudophilotes* switched to Lamiaceae. 

Our study demonstrated that within the subgenus *Pseudophilotes*, only three species, *P. panope, P. abencerragus,* and *P. barbagiae*, were clearly differentiated with respect to DNA barcodes and other studied molecular markers (Figure 1, Figure 2, Figure 3 and Figure 4). As for the species complex *P. baton, P. panoptes, P. vicrama, P. sinaicus*, and *P. jacuticus*, as noted earlier, they share the same or similar DNA barcodes (Figure 4) despite their morphological differences [21]. With the data available, it is impossible to decide whether this complex represents completely separated species with secondary contacts, stages of an incomplete speciation, or a single polymorphic species [21]. In our opinion, in accordance with the principle of nomenclatural stability and preservation of traditionally recognized taxa, *P. baton, P. panoptes, P. vicrama, P. sinaicus*, and *P. jacuticus* should be interpreted as species until further evidence is obtained in favor of or against their species status. In any case, we must state that molecular (based on DNA barcodes) identification of the species *P. baton, P. panoptes, P. vicrama, P. sinaicus*, and *P. jacuticus* seems to be problematic.

Within the *Scolitantides*–*Glaucopsyche* lineage (3), the genus *Glaucopsyche* was revealed in our study to be a paraphyletic group, with the species *Glaucopsyche piasus* forming a separate cluster on the tree (Figure 3). However, support for major basal branches within the *Scolitantides*–*Glaucopsyche* lineage was low in our study; therefore, the identified paraphyly of the genus *Glaucopsyche* cannot be considered proven. The genus *Glaucopshyche* was revealed as a paraphyletic entity in a phylogenomic study by Ugelvig et al. [6] and as a monophyletic group in a whole-genome study by Zhang et al. [9]. The later authors revealed a closer relationship between *Glaucopsyche piasus* (subgenus *Phaedrotes*) and other *Glaucopsyche* species than with *Iolana*, *Praephilotes, Scolitantides,* and *Philotes*. Our data showed that the genus *Glaucopsyche* also included three additional sublineages, which together formed a monophyletic unity. These three lineages can be interpreted as the subgenera *Glaucopsyche* sensu stricto, *Apelles* Hemming, 1931, and *Bajluana* Korshunov and Ivonin, 1990.

Within these three later subgenera, *Bajluana* was the most differentiated with respect to male genitalia [36,55]. The subgenus *Bajluana* included one species, *Glaucopsyche* (*Bajluana*) *argali*, which is endemic to the Altai and Saur-Tarbagatai Mts. Four groups of populations of this species are known: (1) the nominotypical subspecies (*G. argali argali*, mountains surrounding the Chuya steppe in the Russian Altai), (2) subspecies *argali chingiz* Churkin, 2005 (the southern part of the Mongolian Altai, (3) subspecies *argali arkhar* Lukhtanov, 1990 (the Saur, Tarbagatai, and Monrak mountains in Kazakhstan) and (4) the southern part of the Kurchum range in the Kazakhstan Altai (Salkyn-Cheku mountain). The analysis of the DNA barcodes showed that despite the geographical isolation, the first, third, and fourth groups of populations were similar to each other. For the second group of populations, molecular data are not yet available.

*Shijimiaiodes divina* is traditionally assigned to the independent genus *Shijimiaiodes* (and sometimes also to the genus *Sinia* by mistake, see [6]). However, molecular data point to its closeness to the core species of the subgenus *Glaucopsyche* (*Glaucopsyche*). Morphologically, this species is also similar to the typical *Glaucopsyche*, especially to *G. lycormas* [56], which differs in the presence of yellow or reddish spots on the underside of the hindwings. It is obvious that the presence/absence of these yellow or reddish spots is a highly variable characteristic within the subfamily Polyommatinae even on an intra-specific level [13]. Therefore, we support the opinion [9] on the synonymy of *Glaucopsyche* Scudder, 1872 (=*Shijimiaeoides* Beuret, 1958).

The subgenus *Glaucopsyche* also includes two little-known species from Central Asia: *G. charybdis* and *G. laetifica*. Both species inhabit near-water biotopes (riverbanks) in the desert zone, and their caterpillars are associated with licorice (*Glycyrrhiza*) (Fabaceae) [57]. The species are allopatric. *Glaucopsyche charybdis* is found in the basins of the Amu Darya, Zeravshan and Syr Darya (Fergana Valley) rivers. *Glaucopsyche laetifica* is found in the basin of the river Ili and in the downstream of the Syr-Darya River. *Glaucopsyche charybdis* (hind wing underside is gray-brown) and *G. laetifica* (hind wing underside is blue-green) are morphologically well distinguishable, but their DNA barcodes turned out to be similar. From the Dzhungarian Alatau Mts in eastern Kazakhstan, the morph *G. alexis* var. *aeruginosa* is known, resembling *G. laetifica* in color. The DNA barcode data showed that the var. *aeruginosa* was a color variant of *G. alexis* and was not conspecific with *G. laetifica*.

The monophyly of the genus *Iolana* and deep molecular differentiation of its species were revealed. This supports the multi-species concept of this genus [58,59] rather than a mono-species (*I. iolas* [4]) or two-species (*I. iolas* and *O. gigantea* [60]) system. There are no molecular data for two species of this genus *(I. gilgitica* and *I. arjanica*) but judging by the degree of morphological differentiation of their genitalia (59), they are good taxa of the species level. A deep differentiation between the African and Iberian populations attributed to *I. debilitata* was revealed. Perhaps they also represent different species.

## 5. Taxonomic Conclusions

We propose the following taxonomic arrangement of the subtribe Scolitantidina Tutt, 1907

**Subtribe Scolitantidina Tutt, 1907** (= Glaucopsychina Hemming, 1931)

**Genus *Euphilotes* Mattoni, [1978]**

  Subgenus *Euphilotes* (*Euphilotes*) Mattoni, [1978]

  Subgenus *Euphilotes* (*Philotiella* Mattoni, [1978])

**Genus***Phengaris* Doherty, 1891 (=*Maculinea* van Ecke, 1915)

**Genus***Caerulea* Forster, 1938

**Genus***Glaucopsyche* Scudder, 1872

   Subgenus *Glaucopsyche* (*Glaucopsyche*) Scudder, 1872 (=*Shijimiaeoides* Beuret, 1958)

   Subgenus *Glaucopsyche* (*Apelles* Hemming, 1931)

   Subgenus *Glaucopsyche* (*Bajluana* Korshunov & Ivonin, 1990)

   Subgenus *Glaucopsyche* (*Phaedrotes* Scudder, 1876)

**Genus** *Iolana* Bethune-Baker, 1914

**Genus** *Praephilotes* Forster, 1938

**Genus** *Palaeophilotes* Forster, 1938 (no molecular data available)

**Genus** *Scolitantides* Hübner, 1819

**Genus** *Turanana* Bethune-Backer, 1916 (= *Otnjukovia* Zhdanko, [1997]; = *Micropsyche* Mattoni, 1981)

**Genus** *Pseudophilotes* Beuret, 1958

   Subgenus *Pseudophilotes* (*Pseudophilotes*) Beuret, 1958 (=*Inderskia* Korshunov, 2000, **syn. nov**.)

   Subgenus *Pseudophilotes* (*Rubrapterus* Korshunov, 1987)

**Genus** *Sinia* Forster, 1940 (no molecular data available)

**Genus** *Subsolanoides* Koiwaya, [1989] (no molecular data available)

We propose the following taxonomic arrangement of the genera *Pseudophilotes* Beuret, 1958 and *Iolana* Bethune-Baker, 1914

**Genus *Pseudophilotes* Beuret, 1958**

  **Subgenus *Pseudophilotes* (*Pseudophilotes* Beuret, 1958)** (=*Inderskia* Korshunov, 2000, syn. nov.)

*P*. (*P*.) *panope* (Eversmann, 1851) (=*svetlana* Yakovlev, 2003, **syn. nov**.; (=*marina* Zhdanko, 2004, **syn. nov**.)

*P.* (*P.*) *abencerragus* (Pierret, 1837)

*P*. (*P*.) *barbagiae* De Prins & Poorten, 1982

*P*. (*P*.) *panoptes* (Hübner, [1813])

*P*. (*P*.) *baton* (Bergsträsser, [1779])

*P*. (*P*.) *vicrama* (Moore, 1865)

*P*. (*P*.) *jacuticus* Korshunov and Viidalepp, 1980

*P*. (*P*.) *sinaicus* Nakamura, 1975

   *P*. (*P*.) *sinaicus sinaicus* Nakamura, 1975

   *P*. (*P*.) *sinaicus jordanicus* Benyamini, 2000 (no molecular data available)

   **Subgenus *Pseudophilotes* (*Rubrapterus* Korshunov, 1987)**

*P*. (*R*.) *bavius* (Eversmann, 1832)

*P*. (*R*.) *fatma* (Oberthür, 1890)

**Genus *Iolana* Bethune-Baker, 1914**

*I. iolas* (Ochsenheimer, 1816)

*I. debilitata* (Schultz, 1905)

  *I. debilitata debilitata* (Schultz, 1905)

  *I. debilitata farriolsi* de Sagarra, 1930

*I. lessei* Bernardi, 1964

*I. alfierii* Wiltshire, 1948

*I. arjanica* Rose, 1979 (no molecular data available)

*I. kermani* Dumont, 2004

*I. andreasi* (Sheljuzhko, 1919)

*I. gilgitica* (Tytler, 1926) (no molecular data available)

*I. gigantea* (Grum-Grshimailo, 1885)

## Figures and Tables

**Table 1:** List of the studied samples and obtained sequences.

Species	BOLD/Field ID	GeneBank	Gene	Country	Locality
*Glaucopsyche alexis* var. *aeruginosa*	BPALB161-16	OP712325	COI	Israel	Hermon
	BPALB162-16	OP712326		Israel	Hermon
	BPAL2627-14	OP712327		Israel	Beit Jan
	BPAL2522-14	OP712328		Israel	Nahal Trivon
	BPAL3276-16	OP712334		Kazakhstan	Dzhungarian Alatau, Kolbai
	BPAL3274-16	OP712332		Kazakhstan	Kolbai
	BPAL3275-16	OP712333		Kazakhstan	Kolbai
	BPAL3408-16	OP712338		Kazakhstan	Kyzylagash
*Glaucopsyche argali*	LOWAM265-11	OP712339	COI	Kazakhstan	Kurtshum Mts, Salkyn-Cheku
	LOWAM268-11	OP712340		Kazakhstan	Salkyn-Cheku
	LOWAM267-11	OP712341		Kazakhstan	Salkyn-Cheku
	LOWAM266-11	OP712342		Kazakhstan	Salkyn-Cheku
*Glaucopsyche laetifica*	BPAL3283-16	OP712335	COI	Kazakhstan	Ili valley, Koktal
	BPAL3284-16	OP712336		Kazakhstan	Koktal
	BPAL3285-16	OP712337		Kazakhstan	Koktal
*Glaucopsyche melanops*	BPAL3540-16	OP712329	COI	Morocco	Agadir 30.90 N 7.24 W
	BPAL3541-16	OP712330		Morocco	Agadir
	BPAL3546-16	OP712331		Morocco	Agadir
*Iolana alfierii*	BPAL2358-14	OP712343	COI	Israel	Avdat
	BPAL2524-14	OP712348		Israel	Avdat
	BPAL2525-14	OP712349		Israel	Avdat
	BPAL2902-15	OP712350		Israel	Har-A-Negev
	BPAL2359-14	OP712352		Israel	Avdat
*Iolana andreasi*	LOWAM286-11	OP712351	COI	Iran	Shahkuh
*Iolana andreasi khayyami*	BPAL2452-14	OP712346	COI	Iran	Tehran, Polur
	BPAL2453-14	OP712347		Iran	Tehran, Polur
*Iolana kermani*	BPAL2450-14	OP712344	COI	Iran	Kerman, Kuh-e-Segoh
	BPAL2451-14	OP712345		Iran	Kerman
*Praephilotes anthracias*	BPAL3279-16	OP712323	COI	Kazakhstan	Matai
	BPAL3280-16	OP712324		Kazakhstan	Matai
*Pseudophilotes abencerragus*	BPALB525-18	OP644300	COI	Israel	
	BPALB526-18	OP644301		Israel	
	BPALB527-18	OP644302		Israel	
	BPALB528-18	OP644303		Israel	
	BPAL3567-16	OP644314		Morocco	32.5853 N 6.05611 W
*Pseudophilotes bavius*	BPALB030-16	OP644305	COI	Russia	Bashkortostan, 54.89 N 53.646 E
*Pseudophilotes panope*	L2-14	OP679877	ITS2	Kazakhstan	Koibyn
	L2-15	OP679878		Kazakhstan	Koibyn
	L2-16	OP679879		Kazakhstan	Koibyn
	L2-14	OP681138	Wingless	Kazakhstan	Koibyn
	L2-14	OP681135	EF1α	Kazakhstan	Koibyn
	L2-15	OP681136		Kazakhstan	Koibyn
	L2-16	OP681137		Kazakhstan	Koibyn
	L2-14	OP678972	28S	Kazakhstan	Koibyn
	L2-15	OP678973		Kazakhstan	Koibyn
	L2-16	OP678974		Kazakhstan	Koibyn
	BPALB512-18	OP644294	COI	Kazakhstan	Koibyn
	BPALB513-18	OP644295		Kazakhstan	Koibyn
	BPALB514-18	OP644296		Kazakhstan	Koibyn
	BPALB515-18	OP644297		Kazakhstan	Koibyn
	BPALB516-18	OP644298		Kazakhstan	Koibyn
	BPALB517-18	OP644299		Kazakhstan	Koibyn
	BPALB601-19	OP644310		Kazakhstan	25 km NE Atyrau
	BPALB602-19	OP644311		Kazakhstan	21 km NE Atyrau
	BPALB603-19	OP644312		Mongolia	Hovd, Arshantyn Nuru
	BPAL3287-16	OP644315		Kazakhstan	Koibyn
*Pseudophilotes vicrama*	BPALB553-18	OP644304	COI	Israel	
	BPALB284-17	OP644306		Tajikistan	
	BPALB331-17	OP644307		Tajikistan	
	BPALB359-17	OP644308		Tajikistan	
	BPALB470-17	OP644309		Israel	
*Pseudophilotes jacuticus*	BPALB605-19	OP644313	COI	Russia	Yakutia, Yakutsk

**Table 2:** Fragments of DNA sequences used for phylogenetic analysis.

Sequence	Total Length, bp	Number of Variable Sites	Number of Parsimony Informative Sites
*COI*	1497	454	353
*COII*	679	184	116
*EF1a*	1161	238	157
*H3*	327	57	42
*ITS2*	449	104	81
*wg*	369	120	67
*28S*	820	93	65

## Data Availability

All the analyzed DNA sequences are available via the GenBank links provided (Table 1).

## Supplementary Materials

The following supporting information can be downloaded at: www.mdpi.com/article/10.3390/insects13121110/s1, Table S1: The concatenated alignment (28S + COI + H3+ EF1-a + wg + COII + ITS2).
